# A perfused multi-well bioreactor platform to assess tumor organoid response to a chemotherapeutic gradient

**DOI:** 10.3389/fbioe.2023.1193430

**Published:** 2023-05-31

**Authors:** Elisa Marie Wasson, Wei He, Jesse Ahlquist, William Fredrick Hynes, Michael Gregory Triplett, Aubree Hinckley, Eveliina Karelehto, Delaney Ruth Gray-Sherr, Caleb Fisher Friedman, Claire Robertson, Maxim Shusteff, Robert Warren, Matthew A. Coleman, Monica Lizet Moya, Elizabeth K. Wheeler

**Affiliations:** ^1^ Materials Engineering Division, Lawrence Livermore National Laboratory, Livermore, CA, United States; ^2^ Biosciences and Biotechnology Division, Lawrence Livermore National Laboratory, Livermore, CA, United States; ^3^ Department of Surgery, University of California, San Francisco, San Francisco, CA, United States; ^4^ Helen Diller Family Comprehensive Cancer Center, San Francisco, CA, United States; ^5^ Department of Biomedical Engineering, Boston University, Boston, MA, United States; ^6^ Department of Computational Media, University of California Santa Cruz, Santa Cruz, CA, United States; ^7^ UC Davis Comprehensive Cancer Center, Davis, CA, United States

**Keywords:** tumor model, drug transport, flow transport, colorectal (colon) cancer, bioreactor 3D cell culture

## Abstract

There is an urgent need to develop new therapies for colorectal cancer that has metastasized to the liver and, more fundamentally, to develop improved preclinical platforms of colorectal cancer liver metastases (CRCLM) to screen therapies for efficacy. To this end, we developed a multi-well perfusable bioreactor capable of monitoring CRCLM patient-derived organoid response to a chemotherapeutic gradient. CRCLM patient-derived organoids were cultured in the multi-well bioreactor for 7 days and the subsequently established gradient in 5-fluorouracil (5-FU) concentration resulted in a lower IC_50_ in the region near the perfusion channel versus the region far from the channel. We compared behaviour of organoids in this platform to two commonly used PDO culture models: organoids in media and organoids in a static (no perfusion) hydrogel. The bioreactor IC_50_ values were significantly higher than IC_50_ values for organoids cultured in media whereas only the IC_50_ for organoids far from the channel were significantly different than organoids cultured in the static hydrogel condition. Using finite element simulations, we showed that the total dose delivered, calculated using area under the curve (AUC) was similar between platforms, however normalized viability was lower for the organoid in media condition than in the static gel and bioreactor. Our results highlight the utility of our multi-well bioreactor for studying organoid response to chemical gradients and demonstrate that comparing drug response across these different platforms is nontrivial.

## Introduction

Colorectal cancer (CRC) is the third most common cancer in both men and women in the US and the second leading cause of cancer-related death in developed countries (Society, 2023). Although surgery is highly successful for patients with localized and locoregional disease (stages I–III), a total of 60% of CRC patients present with stage IV liver metastasis (CRCLM), for which surgical therapy is not curative. Combination regimes of chemotherapeutic agents and targeted therapies (e.g., vascular endothelial growth factor (VEGF) inhibitors, epidermal growth factor receptor inhibitors) benefit some patients (Martins et al., 2018), however the 5-year relative survival rate for metastatic CRC remains poor at 14%. Thus, there is an urgent need to develop additional therapies for CRCLM.

Patient-derived organoids (PDOs) are emerging as reliable preclinical tools to develop and test therapies for metastatic CRC as they have been shown to phenocopy drug response behavior of the tumor from which they are derived and retain genomic and some phenotypic characteristics of the original tumor (Ooft et al., 2019; Rizzo et al., 2021). While PDOs have enabled great strides in drug development and screening, drug penetration and transport are also important factors that affect treatment efficacy *in vivo* yet are often overlooked in drug screening studies (Wasson et al., 2021). PDOs are often cultured within media or extracellular matrices, typically basement membrane-like gels such as Matrigel, however drug transport differences in these culture conditions are often not characterized, making it difficult to understand the influence of drug transport on treatment outcomes and challenging to compare to *in vivo* studies (Verduin et al., 2021).

Drug transport in tumors *in vivo* is complex. Tumor stromal cells secrete abnormal desmoplastic extracellular matrix that may prohibit transport of drugs to tumors. In addition, tumor-associated blood vessels are often malformed and leaky, creating tortuous pathways for flow and a rise in the interstitial pressure in the center of tumors, driving nutrients and drug molecules out (Jain, 1987; 1999; Munson and Shieh, 2014). Consequently, drug concentration varies throughout the tumor with regions of high and low concentration. Heterogeneity in drug concentration may lead to heterogeneity in treatment and could contribute to treatment resistance and recurrence (Marusyk et al., 2020). To better understand tumor progression and treatment resistance it is necessary to develop *in vitro* platforms that enable microenvironmental engineering with high levels of control over flow patterns and gradients of drugs.

We developed a perfused multi-well bioreactor platform capable of studying CRCLM PDO response to various chemotherapeutic gradients. Our platform, constructed using a standard 12 well tissue culture plate, facilitates easy assembly, eliminates the need for microfabrication techniques, and enables up to 12 independent conditions. Within this device we generated reproducible cylindrical channels for continuous perfusion in a fibrin-gelatin hydrogel using a novel sacrificial maltodextrin molding technique. Using finite element simulation, we characterized transport of the chemotherapeutic agent 5-fluorouracil (5-FU) in the multi-well bioreactor and evaluated organoid response to the resulting drug gradients. We then compared these results to other standard PDO culture conditions (organoid in media and organoids in bulk hydrogel) to investigate the variances in drug transport that occur between these conditions and how these differences subsequently affect organoid response.

## Materials and methods

### Organoid culture

The PDOs used in this experiment were derived from a patient that underwent resection for CRCLM at the University of California, San Francisco in 2016. The research protocol was approved by the institutional review board of UCSF (IRB#10-05031). The tumor specimen obtained during surgery was used to generate a mouse xenograft as previously described (Kondo et al., 2019). Animal experiments were carried out by members of the UCSF Preclinical Therapeutics Core Facility in accordance with the University of California San Francisco animal care and use committee (IACUC# AN179937-03A). Briefly, the tumor was minced with a scalpel and digested with Liberase (Sigma) and DNase (Qiagen) for 1 h at 37°C in a Gentle Macs Octo Dissociator (Miltenyi). The digested specimen was then filtered sequentially through 500 μm, 250 μm, and 100 μm filters to remove undigested tissue. Cell clusters were collected using a 40 μm filter and transferred to ultra-low attachment plates and cultured in organoid media (DMEM/F12 Glutamax (Gibco), 1× PenStrep/Glutamine (Invitrogen), 1× STEMPRO hESC SFM (Invitrogen), 0.1 mM beta-mercaptoethanol, 8 ng/mL bFGF (Invitrogen), 1.8% BSA (Invitrogen) for 5 days prior to cryopreservation using CryoStor CS10 (BioLife Solutions).

**FIGURE 1:**
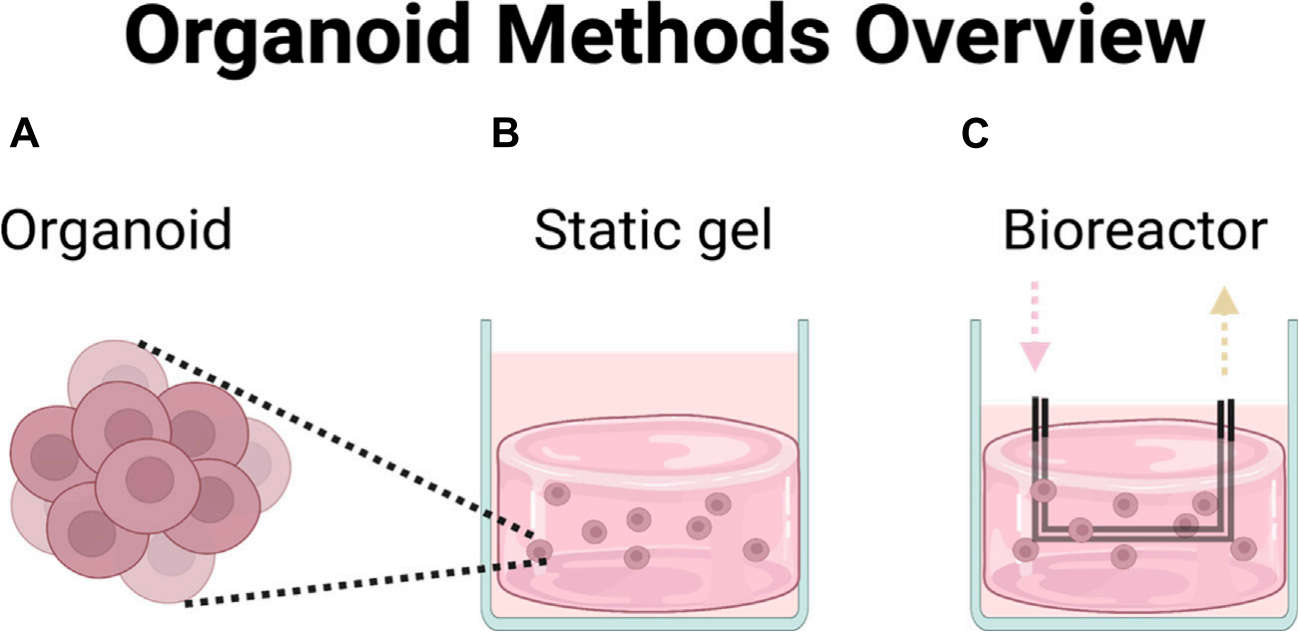
Chemotherapeutic efficacy was compared across three different platforms. **(A)** Organoids in media, **(B)** organoids in hydrogel, **(C)** organoids in perfused multi-well bioreactor platform.

Organoids were thawed, resuspended in phosphate-buffered saline (PBS) (Gibco, 10010023) to wash away DMSO and pelleted by centrifuging at 300 g for 5 minutes. Pellets were then gently resuspended in 10 mL StemPro CRC media comprised of DMEM/F-12 GlutaMAX (Gibco, 10565-018), 1× Antibiotic-Antimycotic (Gibco, 15240062), 1× StemPro hESC Growth supplement (Gibco, A10006-01), 1.8% BSA (Gibco, A10008-01), 8 ng/mL bFGF (Gibco, PHG0261), and 0.1 mM 2-Mercaptoethanol (Gibco, 21985023). Organoids were cultured for 4 days in ultra-low adhesion 10 cm dishes (Corning, 3,262) prior to use in experiments. After 4 days, organoids were filtered through a 40 μm cell strainer (Fisher Scientific, 087711) to eliminate single cells and counted. Organoids were then cultured in three different conditions (Figure 1): organoids in media, in static gel, or in the multi-well bioreactor.

### Organoid culture in media and static gel (no perfusion) conditions

Once organoids were collected and counted, they were subsequently cultured in either media (StemPro CRC media) as described above or in fibrin-gelatin hydrogels for experimental studies with the chemotherapeutic drug 5-Flurouracil (5-FU). Solutions for the fibrin-gelatin hydrogel were all prepared in PBS without Ca^2+^ and Mg^2+^ (Gibco) and mixed, as described below, resulting in a final gel consisting of gelatin (75 mg/mL, type A, 300 Bloom, Sigma-Aldrich), fibrinogen (10 mg/mL; type I-S, Sigma-Aldrich), transglutaminase (TG; 2 mg/mL; type TI, Modern Pantry), CaCl_2_ (2.5 mM, Sigma-Aldrich), and thrombin (1 U/mL; Sigma-Aldrich) (Kolesky et al., 2016; Hynes et al., 2020). 150 mg/mL gelatin stock solutions were prepared in PBS by heating at 70°C for 2 h with gentle stirring until completely dissolved. The gelatin solution was then brought to a pH of 7.5 with NaOH or HCl, then sterile filtered, aliquoted, and stored at 4°C until use. All other materials were prepared the day of experiments, sterile filtered and kept at 37°C until just before mixing.

Fibrinogen, TG, and CaCl_2_ were mixed carefully via pipette and allowed to incubate for 5 min at 37°C. Gelatin was then added to the solution, mixed thoroughly, and allowed to incubate for 20 min at 37°C. Finally, the premixed gel solution was added to thrombin and mixed thoroughly before being added to the organoids to obtain a final organoid concentration of 375 organoids/mL. 1 mL of the organoid and fibrin-gelatin hydrogel solution was dispensed into each of the wells of a 12 well plate. After 10 min at room temperature, 1 mL CRC media was overlaid on the hydrogel in each well and the plate was incubated in a cell culture incubator at 37°C for 1 hour followed by 4°C for 20 min to simulate the conditions required for bioreactor fabrication as described below.

Organoids in media and organoids in static hydrogels were cultured for 1 day prior to addition of 5-FU. 5-FU was selected because it is a common chemotherapeutic agent used in the treatment of metastatic CRC (Biller and Schrag, 2021; Morris et al., 2023). 5-FU was prepared at a stock concentration of 50 mM in DMSO and stored at −20°C before adding to media to result in applied concentrations of 0, 1, 5, 25, 100, 150 μM for organoids in media and 0, 2, 10, 50, 200, and 300 μM for the static gel.

### Bioreactor design and fabrication

**FIGURE 2:**
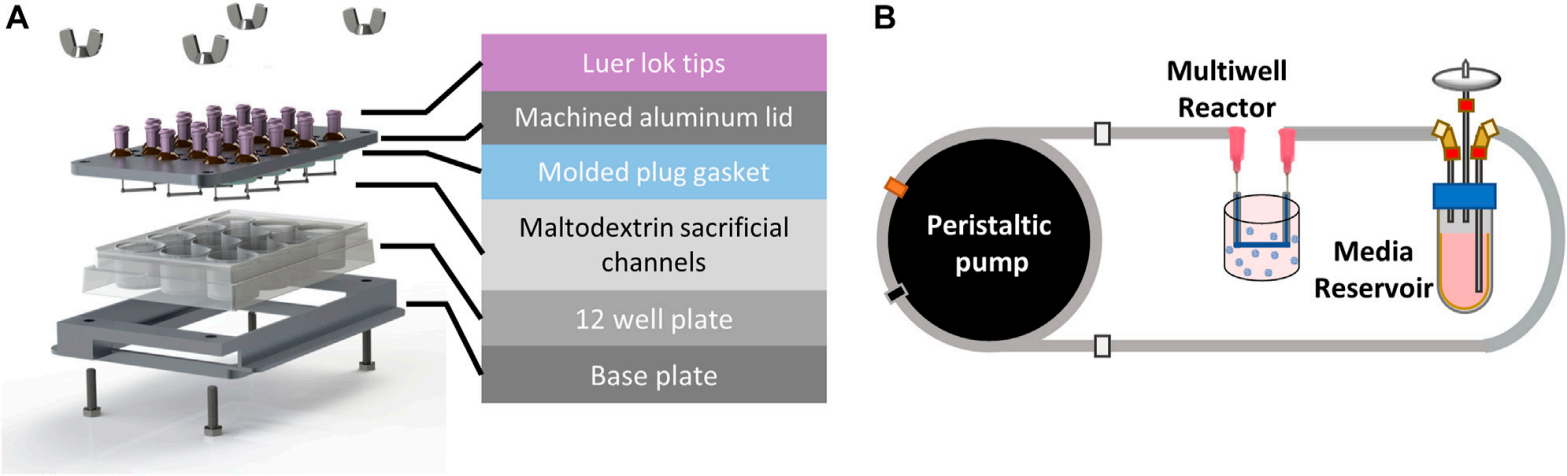
Perfused multi-well bioreactor platform. **(A)** Exploded view showing molded plug gasket, maltodextrin sacrificial channels, and custom machined aluminum lid and base to seal the commercially available 12 well plate. **(B)** A peristaltic pump was used to recirculate organoid media from a reservoir to each well of the bioreactor at a flow rate of 20 μL/min.

Our multi-well bioreactor platform was designed to utilize a standard 12 well tissue culture plate for ease of handling and fabrication (Figure 2A). A custom machined aluminum lid was designed to include fluidic inlet and outlet ports (Nordson stainless steel needles, 20 gauge) for each well and two filling ports per well to allow the user to dispense hydrogel into the wells. A PDMS gasket was fabricated using a custom mold to ensure each well remained fluidically isolated. All bioreactor components (i.e., aluminum base and lid, fluidics, and PDMS gasket) were sterilized by autoclaving prior to use.

Spray dried maltodextrin was molded to form sacrificial channels (Supplementary Figure S1) and sterilized overnight in an oven at 100°C. To assemble the bioreactor, the PDMS gasket was placed onto the underside of the aluminum lid. Sacrificial channels were then attached to the ends of the stainless-steel pins using a 35% (w/w) solution of Pluronic F127 (Sigma) in deionized water. Briefly, the Pluronic solution was loaded into a 1 mL syringe and allowed to solidify at room temperature for 30 min prior to use. A small bead of Pluronic was extruded onto the ends of each pin and maltodextrin channels were gently placed on top using tweezers (Supplementary Figure S2). The Pluronic beads were then allowed to dry for 30 min before flipping the lid over and inserting into the 12 well plate. The bioreactor base and lid were then clamped together using hex bolts and wing nuts (McMaster Carr) until the PDMS gasket was sufficiently and evenly compressed.

Organoids were mixed with the fibrin-gelatin hydrogel at a concentration of 375 organoids/mL as previously described and 5 mL were dispensed into each well to encapsulate the sacrificial channels. The bioreactor was then placed in a humidified incubator with 5% CO_2_ for 1 h at 37°C followed by 20 min at 4°C to allow the Pluronic beads to liquefy before flushing the sacrificial maltodextrin from the channels. Upon completion of the incubation steps, the sacrificial maltodextrin and Pluronic were flushed from each of the wells using 3 mL warm CRC media and the wells were connected to flow.

### Perfusion fluidics setup

A peristaltic pump (MP2, Elemental Scientific) was used to deliver flow to each of the wells from media reservoirs (14 mL tube, Corning) filled with 5 mL of CRC media in a series of fluidic connections (Figure 2B). Media was drawn from the reservoir using 1/16″ PFA fluidic tubing (0.01” ID, IDEX) that was connected to peristaltic tubing (0.13 mm ID, Elemental Scientific) attached to the peristaltic pump, followed by another section of PFA tubing that directly connected to the inlet of the well with a union and luer adapter. Media was returned to the media reservoir from the outlet of the well using fittings and a short section of PFA tubing. The pump was set to 30 rpm which corresponded to a flow rate of 22 μL/min and was verified using a flow sensor (MFS3, Elveflow). 5-FU was added to the media reservoirs after 1 day of culture at nominal concentrations of 0, 4, 20, 100, 400, and 600 μM in 5 mL CRC media. The media and drug solutions were recirculated in each of the wells for the duration of the 7-day experiment.

### Transport simulations

Simulations of chemotherapeutic transport in the bioreactors were conducted using COMSOL Multiphysics 5.5a. The bioreactor was modeled in COMSOL’s native CAD software; Free and Porous Media Flow (fp) and Transport of Diluted Species in Porous Media (tds) modules were used to solve the Navier Stokes and chemical diffusion equations respectively, while the Global ODEs and DAEs (ge) module was employed to model the recirculating inlet and outlet of the bioreactor. Standard no-slip boundary conditions were assumed and drug consumption by tumor organoids was considered negligible. Diffusivity of 5-FU was determined through previously described methods (Hsu et al., 2018) (Supplementary Section 1.2). Hydrogel porosity of 0.9 was determined via the Archimedes Method (Ho and Hutmacher, 2006) and permeability values for the hydrogel regions were taken from our previous work (Alonzo et al., 2015).

A parametric sweep of initial inlet concentrations ranging from 0–600 μM for the 7-day experimental time course was performed. Average drug concentrations for the static gel condition and regions near (region 1) and far (region 2) from the bioreactor channel were evaluated for different time points in COMSOL post-processing to inform the applied 5-FU concentration for each experimental platform with the goal of achieving similar cumulative dose values.

An area under the curve (AUC) metric was calculated by integrating the average concentration in each platform (media, static gel, bioreactor region 1 and 2) simulation over the treatment duration (0–7 days) using a trapezoidal Reimann sum. This metric provided the total 5-FU dose that was delivered to the organoids in each culture condition during the weeklong experiment. For the organoid in media condition, it was assumed that the concentration remained constant (i.e., 5-FU was neither consumed nor added) throughout the duration of the experiment.

### Organoid area and circularity

To determine whether 5-FU concentration influenced organoid size or morphology, organoids in each of the culture conditions (media, *n* = 3; static gel, *n* = 30; bioreactor, *n* = 10) were imaged in brightfield on day 7 of the experiment using an Olympus IX83 microscope fitted with a ×10 objective. Organoid area and perimeter were measured manually using Fiji v1.53s (Schindelin et al., 2012). Circularity was calculated using the following equation $\left(circularity=\frac{4\pi *Area}{{perimeter}^{2}}\right)$ .

### CellTiter-Glo viability and IC_50_ analysis

After 7 days of exposure to 5-FU, cells from each culture modality were collected and assessed for viability using the CellTiter-Glo 3D assay (Promega, G9681) according to the manufacturer’s instructions.

For organoids cultured in media, organoids were spun down and resuspended in StemPro CRC media, 100 μL per well for 96-well. CellTiter-Glo^®^ Reagent was added to each well and contents were mixed for 2 min on an orbital shaker to induce cell lysis. The plate was incubated at room temperature for 10 min to stabilize the luminescent signal and luminescence was recorded using a plate-reading luminometer (SpectraMax M5e, Molecular Devices).

For organoids cultured in the static gel, a sterile scalpel and sterile forceps were used to mince and transfer the cell laden gels into a sterile 5 mL syringe barrel. The gels were then fully homogenized by pushing the gel through the syringe (no needle attached) into a 15 mL conical tube. A 0.2% collagenase I (Gibco, 17100-017) solution was added to the conical tube and allowed to agitate on a shaker for 1 h at 37°C and 200 rpm to fully dissolve the gel. The solution was then spun down and the pellet collected for use in the CellTiter-Glo assay as previously described.

For the bioreactor samples, the bioreactor gel was cut into equal volumes near and far from the channel (region 1 and 2 respectively). The gel was first cut along the length of the channel to make two-half circles, then each piece was cut parallel to the channel at a distance of 4.5 mm from the channel. Each of the pieces was further cut into two along the symmetry axis to create a total of 4, equally weighted replicates for each region. Once cut, the gels were homogenized and dissociated using the protocol described above for the static gel conditions.

All the CellTiter-Glo results were plotted against the average concentration on day 7 for each condition and IC_50_s calculated using GraphPad 8.0 (Prism) using nonlinear regression. All IC_50_ curves were normalized using the lowest and highest concentration of drug.

### LactateGlo viability analysis

Samples (100 μL) from the supernatant of organoids cultured in media and static gels or from the media reservoirs of the bioreactor were taken at days 0, 2, 4, and 7. The LactateGlo assay (Promega, J5021) was performed on these samples according to the manufacturer’s instruction. Briefly, 50 μL of collected media sample or lactate standard was transferred into a 96-well plate and 50 μL of Lactate Detection Reagent was added. The plate was shaken for 30–60 s and subsequently incubated for 60 min at room temperature. Luminescence was recorded using a plate-reading luminometer (SpectraMax M5e, Molecular Devices) and converted to lactate concentrations using the standard curve. Lactate concentrations were normalized to the lowest and highest 5-FU concentrations and EC_50_ curves were calculated using GraphPad 8.0 (Prism) using nonlinear regression.

### Statistical analysis

Multiple linear regression was used to test if culture platform and 5-FU dose correlated with organoid area or circularity. Multiple linear regression was also used to test if viability analysis with the CellTiter-Glo assay correlated with lactate analysis using the LactateGlo assay (Supplementary Figure S6). For all Lactate-Glo results and organoid area/circularity for bioreactor regions 1 and 2 (Supplementary Figure S4), a two-way ANOVA was performed with Tukey *post hoc* comparisons. IC_50_ curves were compared using their confidence intervals. If confidence intervals did not overlap, the results were considered statistically significant. In the case that they did overlap, an extra sum-of-squares F test was used to determine if IC_50_ values differed between groups. All statistical tests were performed with a *p*-value of 0.05.

## Results

### Multi-well bioreactor design and fabrication

Our multi-well bioreactor platform was designed around a standard 12-well cell culture plate to minimize the number of custom parts and enable facile assembly. This layout provides 12 independently fed wells that can be used to study multiple drug concentrations or combinations in one experiment. To further advance our goal of facile assembly, we developed a method to precisely mold sacrificial maltodextrin, eliminating the need for microfabrication steps often required to produce fluidic channels. Channels formed using molded sacrificial maltodextrin had highly reproducible diameters (2,272 ± 167 μm) ensuring consistent flow with each experiment (Supplementary Figure S2B). A custom CNC machined aluminum lid was used to easily connect each well of the bioreactor to a peristaltic pump that recirculated flow to each of the wells, enabling long-term studies and sampling from connected media reservoirs (Figure 2).

### The transport of 5-fluorouracil in the media, static gel, and bioreactor conditions

**FIGURE 3:**
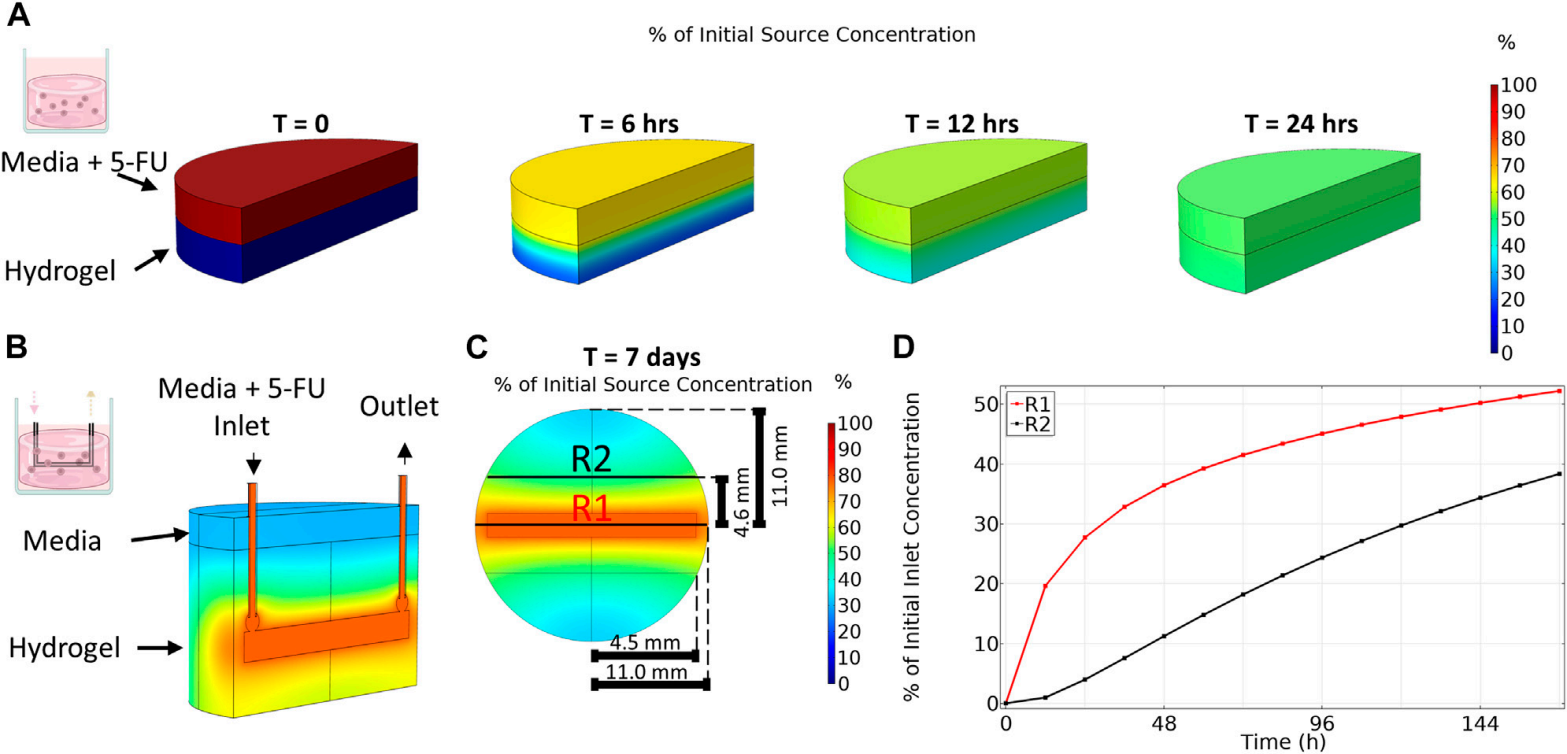
Transport of 5-Fluorouracil varies between platforms. **(A)** Transport of 5-Fluorouracil for organoid in gel condition (no flow) results in a homogenous concentration after 24 h. **(B)** An isometric view cut along length of channel shows that transport of 5-Fluoruracil in the multi-well bioreactor results in a gradient in drug concentration even after 7 days. **(C)** Top-down view taken at channel center. **(D)** Region 1 (R1), close to the bioreactor channel, has a higher average concentration over time compared to region 2 (R2) which is far from the channel.

Drug concentration in the organoid in media condition was assumed to be constant and the effective average concentration equal to the applied concentration for the duration of the experiment (0, 1, 5, 25, 100, 150 μM). To predict the distribution of 5-FU within our platforms and select the applied drug concentrations, the dynamics of transport of 5-FU were simulated in the static gel and bioreactor platforms. In the static gel, 5-FU diffused quickly from media into the gel, reaching steady state at 24 h of culture (Figure 3A). Since the volumes of gel and media were equal in this condition, the resulting steady state concentration is exactly 50% of the source concentration homogenously spread throughout the gel and media. Informed by these results, we applied a concentration 2× higher than the organoid in media condition (0, 2, 10, 50, 200, 300 μM) to achieve a similar cumulative dose for the duration of the experiment.

In contrast to the static gel condition, even after 7 days of culture, drug concentration in the bioreactor had not reached steady state and a gradient in 5-FU concentration remained (Figure 3B) where 5-FU concentration varied with distance from the channel. As expected, region 1, closest to the channel, contained a higher concentration of 5-FU during the entire 7-day period than region 2, further from the channel (Figure 3C). The disparity in drug concentration peaked early then narrowed over time. After 7 days, region 1 reached an average concentration of 52% of the applied 5-FU concentration whereas the average concentration in region 2 was only 38% of the applied concentration. At day 7, the total average concentration in the bioreactor was 45% of the applied concentration. Since the simulation showed diffusion occurred much slower in the bioreactor, we applied a concentration 4× higher than the media condition (0, 4, 20, 100, 400, 600 μM) to achieve a similar cumulative dose between platforms over the duration of the experiment.

### Organoid area and circularity

**FIGURE 4:**
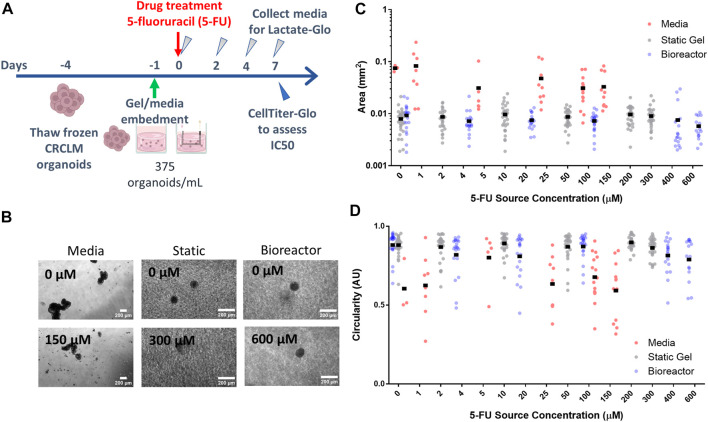
Organoid morphology and size does not change with drug concentration. **(A)** Experimental timeline and process. **(B)** Organoids at the lowest and highest drug concentrations in all three platforms for day 7. Organoid area **(C)** and circularity **(D)** for all platforms.

To investigate how tumor organoids respond to 5-FU in varying environments, we cultured patient-derived colorectal liver metastasis organoids in three different platforms: media, static gel, and a multi-well bioreactor platform (Figure 4A). Organoids cultured in media formed large irregularly shaped clusters over the duration of the experiment whereas organoids cultured in the static gel or multi-well bioreactor were significantly smaller and remained single and more circular (Figure 4B).

For the organoid in media condition, organoid area tended to decrease with increasing dose (with a statically significant nonlinear regression coefficient of −1.1e-4 mm^2^/drug concentration, *p* < 1e-7, Figure 4C) whereas in the static gel and bioreactor conditions, area tended to increase slightly with 5-FU dose (regression coefficients of 1.1e4 and 1.1e4 respectively, *p* < 1e-6, Figure 4C).

No statistically significant differences were found when comparing organoid area or circularity for region 1 and region 2 of the bioreactor (Supplementary Figure S4).

### Cell viability analysis

**FIGURE 5:**
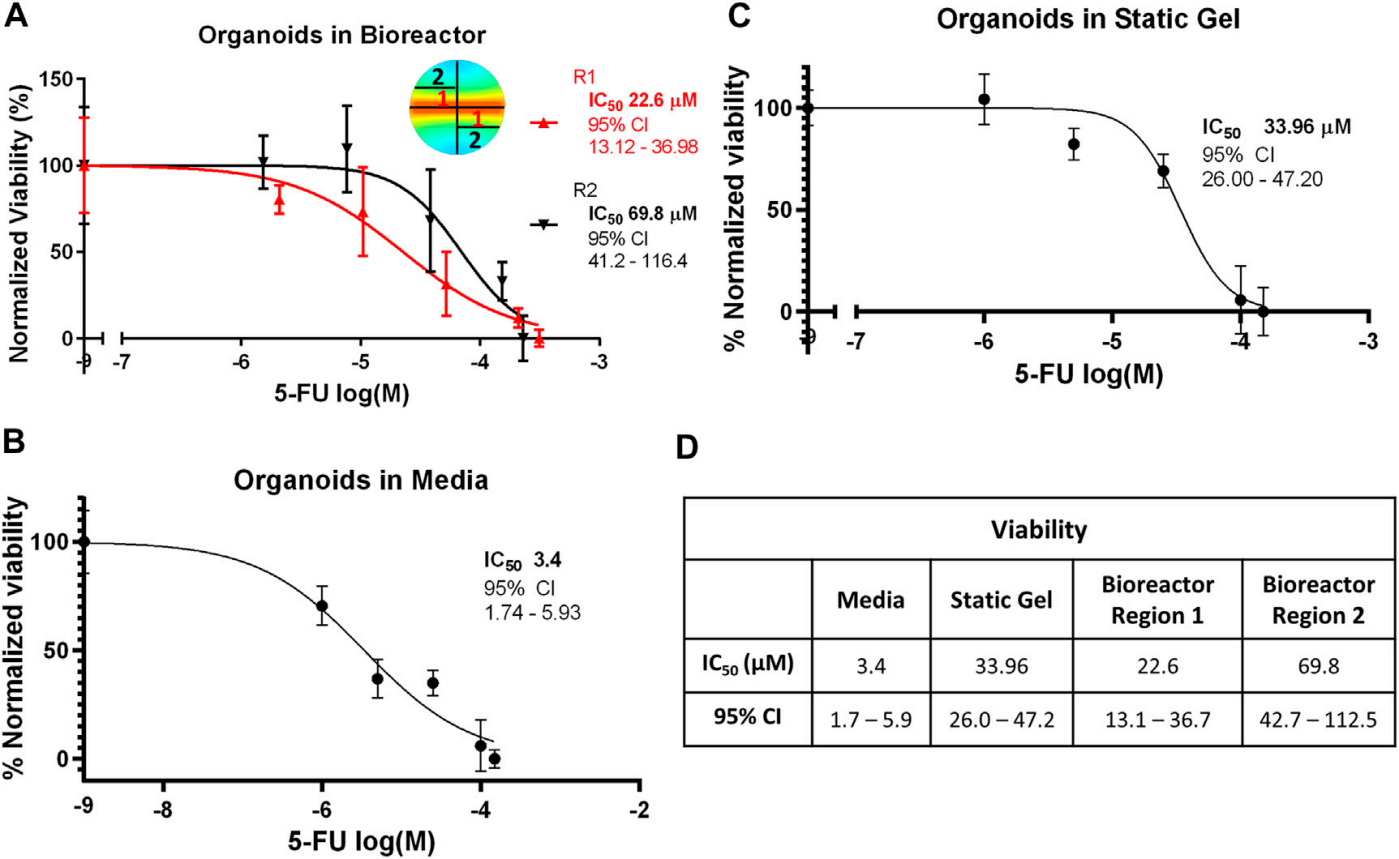
IC_50_ curves for the **(A)** bioreactor, **(B)** static gel, and **(C)** organoid in media conditions determined using the CellTiter-Glo assay. **(D)** IC_50_ values for all culture conditions.

To evaluate whether the gradient in 5-FU concentration in the bioreactor predicted by the transport simulation would alter organoid response to 5-FU, the bioreactor gel was divided into equally weighted sections on day 7 that were located close to the channel (region 1) and far from the channel (region 2). The CellTiter-Glo assay was performed and IC_50_ curves were plotted for both regions (Figure 5A). Our modeling showed that region 1 experienced a higher average 5-FU concentration than region 2 and concomitantly, we observed a statistically significant lower IC_50_ value in region 1 (Figure 5D). The IC_50_ for the organoid in media condition (3.387 μM) was an order of magnitude lower and statistically significantly different than the static gel condition and both regions of the multi-well bioreactor (Figures 5B, C). The IC_50_ for the static gel condition (33.96 μM) was comparable to region 1 of the bioreactor (22.6 μM, *p* = 0.1413) but was significantly lower than region 2 (69.8 μM, *p* = 0.0184).

### Lactate secretion analysis

**FIGURE 6:**
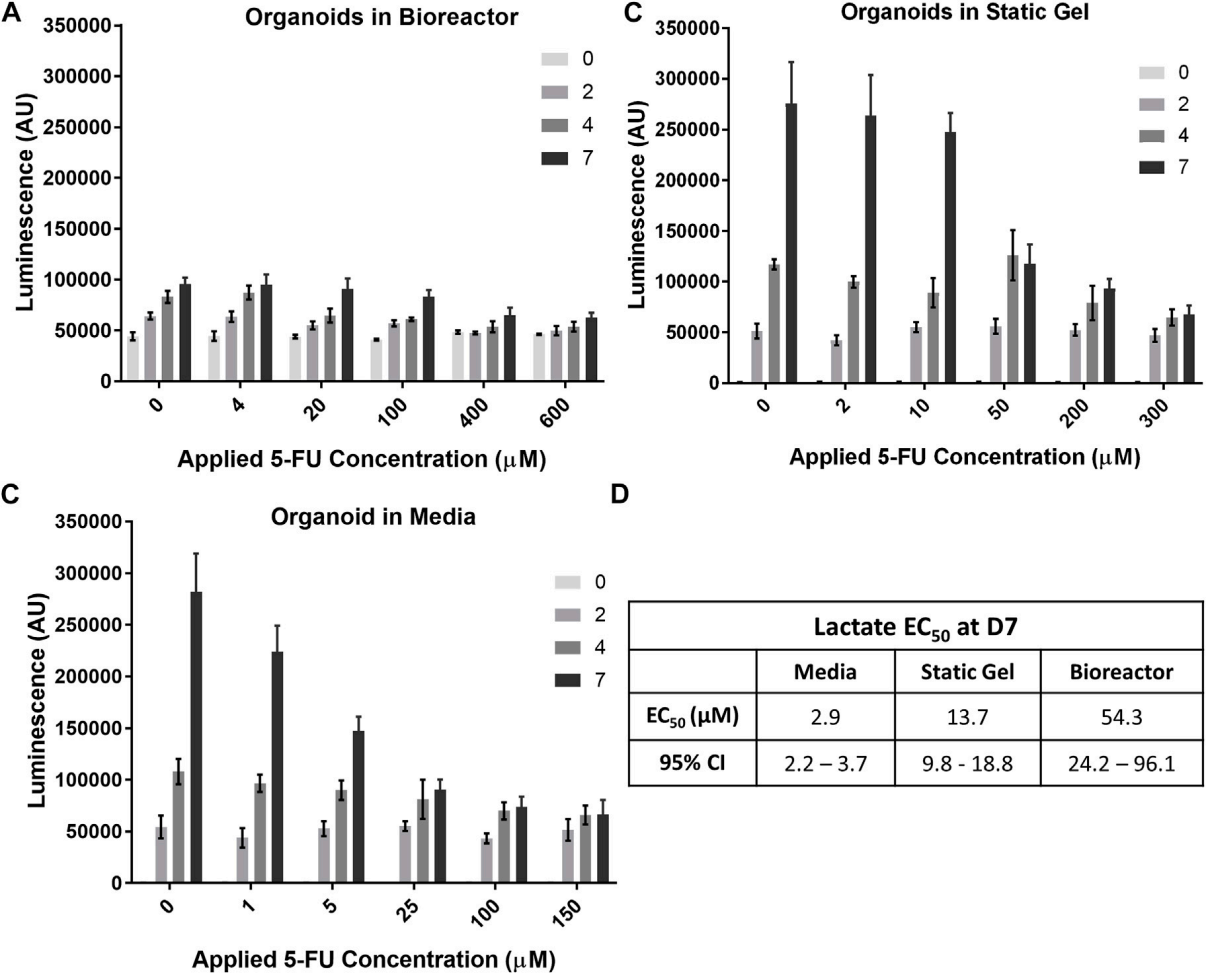
Temporal organoid response to 5-FU can be determined in a nondestructive manner using the LactateGlo assay. Lactate secretion from organoids is dose dependent over time in the **(A)** bioreactor, **(B)** static gel, and **(C)** media conditions. **(D)** EC_50_ values for all culture conditions.

The temporal response of organoids to 5-FU was evaluated using the non-destructive Lactate-Glo assay. As expected, at time 0, there was no statistically significant difference in lactate secretion for the range of 5-FU concentrations (Figure 6A) in the multi-well bioreactor. However, changes in metabolic output could be seen starting at day 2, with significantly less lactate secretion in either of the two highest 5-FU dosed conditions compared to the two lowest 5-FU conditions (*p* < 0.05), with increasing separation over time (*p* < 0.001, days 4&7). Lactate secretion continued to increase for all 5-FU concentrations in the bioreactor between days 4 and 7 of the experiment, however differences were not statistically significantly (400 μM, *p* = 0.059; 600 μM, *p* = 0.18). Overall lactate levels were lower in the bioreactor than in the media and static gel conditions. Using the Lactate-Glo data taken at day 7 and a standard curve, it was possible to derive an EC50 curve which resulted in an EC50 of 54.33 μM (Figure 6D; Supplementary Figure S5).

Lactate levels in the organoid in media and static gel conditions (Figures 6B, C) decreased significantly with increasing dose at day 7 but remained similar at day 2 with inhibition seen only for the two highest concentrations on day 4 (*p* < 0.05). Interestingly, on day 7, lactate levels for the organoids in media at a 5 μM applied concentration separated further from the lower concentrations (0 μM and 1 μM) than the static gel at a 10 μM applied concentration despite experiencing similar cumulative doses for most of the experiment indicating improved cell inhibition (Figures 6B, C). The EC50 derived from Lactate-Glo measurements for organoids in media (2.87 μM), static gel (13.65 μM), and the multi-well bioreactor (54.33 μM) were all statistically significant from one another (Figure 6D).

Regression analysis showed that there is a significant correlation between CellTiter-Glo (C) and LactateGlo (L) data for the media condition (9 units L/C, *p* < 1e-5), but not static gel conditions (4 L/C, *p* = 0.07) or the bioreactor condition (−6 L/C, *p* = 0.07).

### Total dose delivered (area under the curve)

**FIGURE 7:**
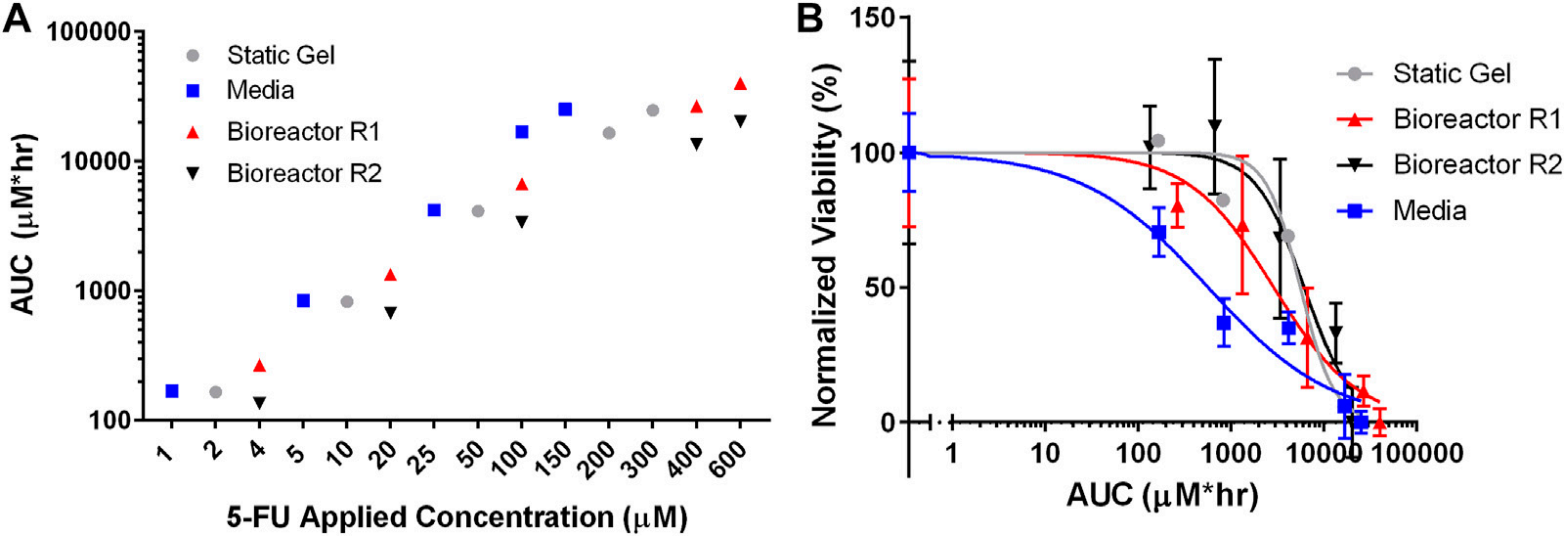
**(A)** Total 5-FU delivered (AUC) by dose and culture method calculated using average concentration over time from the simulations. **(B)** IC_50_ curves for all culture conditions normalized for total 5-FU delivered (AUC).

To compare drug transport more directly between the three culture conditions, the area under the curve (AUC) was calculated by integrating the average concentration from the transport simulations over the duration of the experiment. Figure 7A shows that each of the culture conditions have comparable cumulative doses for each set of applied concentrations. For the lowest applied concentrations (media—1 μM, static gel—2 μM, bioreactor—4 μM), the AUCs were 168 μM*hr, 165 μM*hr, 267 μM*hr, and 135 μM*hr for media, static gel, bioreactor regions 1 and 2 respectively. Bioreactor region 1 had a higher AUC than region 2 since it was closer to the perfused channel. At the same applied concentration (100 μM), the organoid in media condition had a higher AUC than bioreactor region 1 and 2 showing that the same applied concentration results in different cumulative doses between platforms, highlighting the need to account for transport differences between platforms when comparing drug response.

Figure 7B showed that the static gel and bioreactor region 2 have overlapping normalized viability curves when plotted against AUC. Bioreactor region 1 had a lower viability curve due to its higher cumulative dose as evidenced by a higher AUC. Despite having similar AUC values, the media condition had a lower normalized viability curve than the static gel and bioreactor conditions.

## Discussion

Patient-derived organoids (PDOs) are emerging as an important form of preclinical drug screening studies as they retain characteristics of the original tumor such as intratumor heterogeneity and have shown to predict *in vivo* drug response (Weeber et al., 2015; Zheng et al., 2020). However, PDOs lack key features of the tumor microenvironment which have shown to influence tumor progression and treatment such as extracellular matrix (Lu et al., 2012; Crotti et al., 2017; Cox, 2021) and flow (Polacheck et al., 2011; Munson and Shieh, 2014; Follain et al., 2020). Therefore, if PDOs are to be used as patient avatars for personalized medicine, it is necessary to develop platforms that incorporate these features, can screen a range of conditions, and adequately characterize drug transport to properly correlate findings to *in vivo* results.

Our multi-well bioreactor platform demonstrates the ability to generate and study drug gradients and their effects on PDOs in a biologically relevant system with a perfusable extracellular matrix. Finite element modeling verified that after 7 days of exposure to the chemotherapeutic agent 5-FU in the bioreactor, a concentration gradient formed, reaching a higher average concentration close to the channel and a lower average concentration far from the channel. This gradient in concentration of chemotherapeutic correlated with a lower IC_50_ in region 1 than region 2.

To complement this viability assay, we also evaluated organoid response over time, using lactate analysis on media samples taken at different time points throughout the experiment. While this assay provided insight into the growth of organoids over time in a non-destructive manner, the relationship between viability and lactate analysis varied between the three platforms we evaluated. Lactate secretion was much lower in the bioreactor condition compared to media and static gel which may indicate diffusion limitations of lactate to the media reservoirs from the hydrogel. Lactate analysis should therefore be paired with other assays that are not impacted by diffusion. Additionally, nutrients such as glucose (180.156 g/mol) or growth factors that have comparable or larger molecular weights to 5-FU (130.078 g/mol) may experience similar hindered transport in the bioreactor which may lead to altered metabolic activity. In future work it will be important to simulate nutrient transport in the bioreactor condition to assess how it influences metabolic activity and drug response.

While microfluidic and bioreactor platforms are emerging as important tools in preclinical drug screening studies (Park et al., 2019), it remains more common to culture PDOs in media or solubilized basement membrane hydrogels such as Matrigel which lack flow. We thus directly compared our bioreactor system to more traditional static hydrogel or suspension culture systems. While all three culture conditions had comparable AUCs for each set of applied concentrations, the organoid in media condition had a lower normalized viability. This result suggests that cumulative dose alone does not explain differences in cell viability for these platforms and that culturing PDOs in a 3D hydrogel influences drug response. Organoids in media were immediately exposed to 5-FU while the static gel and bioreactor were exposed slowly as the drug diffused through the hydrogel. This time delay in drug exposure may have contributed to this differential organoid response. Additionally, while our model captured diffusion kinetics it did not account for absorption of drug in the hydrogel or other phenomenon that may reduce effective drug concentration. The architecture and constituents of extracellular matrix (ECM) are known to play a critical role in tumor progression and may influence drug response (Henke et al., 2019). Our results demonstrate that comparing drug response across these different platforms is nontrivial; differences in feeding and diffusion dramatically affect expected drug exposure and consequently organoid response.

The static gel and region 2 of the bioreactor had similar AUCs and normalized viability, however, our bioreactor platform generates a gradient of drug concentrations in one platform, a key advantage over the static gel model. Introducing drugs through a perfusable channel enables the study of organoid response to a temporally and spatially developing drug concentration which is not possible in the static gel condition as it reaches an equilibrium condition after only 24 h while the bioreactor maintains a gradient up to 7 days. Additionally, the static gel condition lacks flow, a key feature of the tumor microenvironment that drives tumor progression and influences drug response (Jain, 1987; 1999; Munson and Shieh, 2014; Marusyk et al., 2020). While the average velocity within the bioreactor hydrogel (0.54 nm/s) does not replicate physiologic interstitial flow within tumors (0.6–1 μm/s) (Jain, 1987), it highlights the capability of our combined bioreactor platform and transport simulations to study a range of flow conditions and their effects on drug transport and organoid response that is not possible in either the static gel or media conditions.

While our work uses an ECM hydrogel, we focus on the influence of transport gradients rather than individual ECM components on organoid response. CRCLM tumors contain multiple matrix proteins many of which are known to affect biological function: we chose to focus on a fibrin-gelatin mix as this has been shown to have optimal curing kinetics for bioprinting efforts and supports cell survival (Kolesky et al., 2016; Dubbin et al., 2020; Hynes et al., 2020; Jang et al., 2020). Additional ECM components native to CRCLM such as collagen and fibronectin, can be incorporated into this hydrogel for future studies (Naba et al., 2014; van Huizen et al., 2019; Kim et al., 2021).

It is important to note that extracellular matrix composition and organization can change drug transport kinetics within a hydrogel. The diffusion coefficients of molecules through hydrogels are similar to diffusion coefficients calculated in water, but importantly, small changes in diffusivity can dramatically change delivery over long time periods. Unlike previous work, we experimentally determined the diffusion coefficient of 5-FU in our fibrin-gelatin hydrogel and used this value in our transport simulations (Ayuso et al., 2019; Moon et al., 2020). This step is necessary to obtain accurate simulation results and therefore key to understanding the role drug transport plays in organoid response.

We recognize that there are limitations to our multi-well bioreactor platform. The channels generated in our platform are relatively large in comparison to capillary vessels found in tumors (10–50 μm) (Jain, 1999). Reproducible channel sizes at the scale of capillary vessels are challenging and will be dependent on the fabrication limitations of our compression mold as well as maltodextrin particle size and packing dynamics. While our vessel sizes are not capillary-like, we are able to generate larger channels in a reproducible manner allowing us to accurately quantify drug transport in our platform using our computational model, a factor that is often overlooked in drug studies. Future work will aim to reduce channel diameter and compare experimental results with computational modeling to determine the effect of channel size on drug transport in our platform and evaluate how this compares to *in vivo* values. While we generated gradients in drug concentration in our platform using a single channel, tortuous and leaky tumor vasculature and associated transport *in vivo* results in a much more complex diffusion landscape (Dewhirst and Secomb, 2017). Our maltodextrin molding technique offers rapid, facile fabrication of hydrogels with channels, and may be used to fabricate more complex channels (Kinstlinger et al., 2020), however the fragility of maltodextrin may make it challenging to remove the molded branched networks from the mold without fracture. Additionally, our bioreactor platform requires a large volume of organoid-hydrogel suspension in comparison to microfluidic devices making it more suitable for use with samples that can be easily expanded like PDOs rather than cells or clinical specimens that may be difficult to source. However, it does not require microfabrication techniques and provides sufficient sample to perform standard biological assays which can be challenging in microfluidic devices. Lastly, although the opaque nature of the aluminum lid makes imaging the organoids over time challenging, effluent sampling allowed us to evaluate temporal organoid response to treatment. Future designs of this system could substitute an optically clear lid to better facilitate real-time monitoring of the system via microscopy techniques.

Despite its limitations, our multi-well bioreactor provides a platform that can be used to investigate organoid behavior under dynamic gradients without the need for complex microfabrication and provides sufficient sample for use of standard biological assays. While in this study we used 5-FU to demonstrate chemotherapeutic concentrations in our platform, in future studies could investigate how environmental gradients such as oxygen or soluble factors from cancer associated fibroblasts (CAFs) influence PDO drug response. Using transport modeling, flow parameters may be tuned to control these gradients and enable testing of a range of therapeutic agents and different dosing strategies. In addition, utilizing spatially controllable methods such as bioprinting, organoids can be placed precisely in desired locations within the bioreactor. CAFs or other stromal cells may also be incorporated directly into the bioreactor hydrogel to study the effects of co-culture on drug response under gradient conditions. Furthermore, our bioreactor system can also be used to study the interaction of direct toxic effects of drugs like 5-FU on vascular endothelium as it has shown to reduce nitric oxide synthase and provoke artery vasospasm and vasoconstriction in the coronary arteries (Alter et al., 2006). Endothelial cells lining vessels restrict extravasation of drugs therefore by tuning the flow rates of endothelial-lined channels, we could mimic physiological shear-stress values or use shear stress values that can cause pathological activation of endothelial cells. Finally, tumor-immune interactions may be studied by circulating immune cells in the channel and observing extravasation and invasion into the surrounding organoid loaded hydrogel.

## Conclusion

We have developed a multi-well bioreactor platform capable of generating gradient conditions and testing multiple drug concentrations on PDOs at once. Our study highlights the importance of properly assessing culture environment to obtain accurate interpretation of drug response using transport simulations and careful selection of viability assays capable of capturing spatially and temporally varying organoid response.

## Data Availability

The raw data supporting the conclusion of this article will be made available by the authors, without undue reservation.

## References

[B1] Alonzo L. F. Moya M. L. Shirure V. S. George S. C. (2015). Microfluidic device to control interstitial flow-mediated homotypic and heterotypic cellular communication. Lab. Chip 15 (17), 3521–3529. 10.1039/c5lc00507h 26190172 PMC4855298

[B2] Alter P. Herzum M. Soufi M. Schaefer J. R. Maisch B. (2006). Cardiotoxicity of 5-fluorouracil. Cardiovasc Hematol. Agents Med. Chem. 4 (1), 1–5. 10.2174/187152506775268785 16529545

[B3] Ayuso J. M. Virumbrales-Munoz M. McMinn P. H. Rehman S. Gomez I. Karim M. R. (2019). Tumor-on-a-chip: A microfluidic model to study cell response to environmental gradients. Lab. Chip 19 (20), 3461–3471. 10.1039/c9lc00270g 31506657 PMC6785375

[B4] Biller L. H. Schrag D. (2021). Diagnosis and treatment of metastatic colorectal cancer: A review. JAMA 325 (7), 669–685. 10.1001/jama.2021.0106 33591350

[B5] Cox T. R. (2021). The matrix in cancer. Nat. Rev. Cancer 21 (4), 217–238. 10.1038/s41568-020-00329-7 33589810

[B6] Crotti S. Piccoli M. Rizzolio F. Giordano A. Nitti D. Agostini M. (2017). Extracellular matrix and colorectal cancer: How surrounding microenvironment affects cancer cell behavior? J. Cell Physiol. 232 (5), 967–975. 10.1002/jcp.25658 27775168

[B7] Dewhirst M. W. Secomb T. W. (2017). Transport of drugs from blood vessels to tumour tissue. Nat. Rev. Cancer 17 (12), 738–750. 10.1038/nrc.2017.93 29123246 PMC6371795

[B8] Dubbin K. Robertson C. Hinckley A. Alvarado J. A. Gilmore S. F. Hynes W. F. (2020). Macromolecular gelatin properties affect fibrin microarchitecture and tumor spheroid behavior in fibrin-gelatin gels. Biomaterials 250, 120035. 10.1016/j.biomaterials.2020.120035 32334200

[B9] Follain G. Herrmann D. Harlepp S. Hyenne V. Osmani N. Warren S. C. (2020). Fluids and their mechanics in tumour transit: Shaping metastasis. Nat. Rev. Cancer 20 (2), 107–124. 10.1038/s41568-019-0221-x 31780785

[B10] Henke E. Nandigama R. Ergün S. (2019). Extracellular matrix in the tumor microenvironment and its impact on cancer therapy. Front. Mol. Biosci. 6, 160. 10.3389/fmolb.2019.00160 32118030 PMC7025524

[B11] Ho S. T. Hutmacher D. W. (2006). A comparison of micro CT with other techniques used in the characterization of scaffolds. Biomaterials 27 (8), 1362–1376. 10.1016/j.biomaterials.2005.08.035 16174523

[B12] Hsu H. H. Kracht J. K. Harder L. E. Rudnik K. Lindner G. Schimek K. (2018). A method for determination and simulation of permeability and diffusion in a 3D tissue model in a membrane insert system for multi-well plates. J. Vis. Exp. 132, 56412. 10.3791/56412 PMC5931342 29553546

[B13] Hynes W. F. Pepona M. Robertson C. Alvarado J. Dubbin K. Triplett M. (2020). Examining metastatic behavior within 3D bioprinted vasculature for the validation of a 3D computational flow model. Sci. Adv. 6 (35), eabb3308. 10.1126/sciadv.abb3308 32923637 PMC7449690

[B14] Jain R. K. (1987). Transport of molecules in the tumor interstitium: A review. Cancer Res. 47 (12), 3039–3051. 3555767

[B15] Jain R. K. (1999). Transport of molecules, particles, and cells in solid tumors. Annu. Rev. Biomed. Eng. 1, 241–263. 10.1146/annurev.bioeng.1.1.241 11701489

[B16] Jang L. K. Alvarado J. A. Pepona M. Wasson E. M. Nash L. D. Ortega J. M. (2020). Three-dimensional bioprinting of aneurysm-bearing tissue structure for endovascular deployment of embolization coils. Biofabrication 13 (1), 015006. 10.1088/1758-5090/abbb9b 32977323

[B17] Kim M. S. Ha S. E. Wu M. Zogg H. Ronkon C. F. Lee M. Y. (2021). Extracellular matrix biomarkers in colorectal cancer. Int. J. Mol. Sci. 22 (17), 9185. 10.3390/ijms22179185 34502094 PMC8430714

[B18] Kinstlinger I. S. Saxton S. H. Calderon G. A. Ruiz K. V. Yalacki D. R. Deme P. R. (2020). Generation of model tissues with dendritic vascular networks via sacrificial laser-sintered carbohydrate templates. Nat. Biomed. Eng. 4 (9), 916–932. 10.1038/s41551-020-0566-1 32601395

[B19] Kolesky D. B. Homan K. A. Skylar-Scott M. A. Lewis J. A. (2016). Three-dimensional bioprinting of thick vascularized tissues. Proc. Natl. Acad. Sci. U. S. A. 113 (12), 3179–3184. 10.1073/pnas.1521342113 26951646 PMC4812707

[B20] Kondo J. Ekawa T. Endo H. Yamazaki K. Tanaka N. Kukita Y. (2019). High-throughput screening in colorectal cancer tissue-originated spheroids. Cancer Sci. 110 (1), 345–355. 10.1111/cas.13843 30343529 PMC6317944

[B21] Lu P. Weaver V. M. Werb Z. (2012). The extracellular matrix: A dynamic niche in cancer progression. J. Cell Biol. 196 (4), 395–406. 10.1083/jcb.201102147 22351925 PMC3283993

[B22] Martins M. Mansinho A. Cruz-Duarte R. Martins S. L. Costa L. (2018). Anti-EGFR therapy to treat metastatic colorectal cancer: Not for all. Adv. Exp. Med. Biol. 1110, 113–131. 10.1007/978-3-030-02771-1_8 30623369

[B23] Marusyk A. Janiszewska M. Polyak K. (2020). Intratumor heterogeneity: The rosetta stone of therapy resistance. Cancer Cell 37 (4), 471–484. 10.1016/j.ccell.2020.03.007 32289271 PMC7181408

[B24] Moon H. S. Yoo C. E. Kim S. Lee J. E. Park W. Y. (2020). Application of an open-chamber multi-channel microfluidic device to test chemotherapy drugs. Sci. Rep. 10 (1), 20343. 10.1038/s41598-020-77324-3 33230163 PMC7683738

[B25] Morris V. K. Kennedy E. B. Baxter N. N. Benson A. B. 3rd Cercek A. Cho M. (2023). Treatment of metastatic colorectal cancer: ASCO guideline. J. Clin. Oncol. 41 (3), 678–700. 10.1200/jco.22.01690 36252154 PMC10506310

[B26] Munson J. M. Shieh A. C. (2014). Interstitial fluid flow in cancer: Implications for disease progression and treatment. Cancer Manag. Res. 6, 317–328. 10.2147/cmar.s65444 25170280 PMC4144982

[B27] Naba A. Clauser K. R. Whittaker C. A. Carr S. A. Tanabe K. K. Hynes R. O. (2014). Extracellular matrix signatures of human primary metastatic colon cancers and their metastases to liver. BMC Cancer 14, 518. 10.1186/1471-2407-14-518 25037231 PMC4223627

[B28] Ooft S. N. Weeber F. Dijkstra K. K. McLean C. M. Kaing S. van Werkhoven E. (2019). Patient-derived organoids can predict response to chemotherapy in metastatic colorectal cancer patients. Sci. Transl. Med. 11 (513), eaay2574. 10.1126/scitranslmed.aay2574 31597751

[B29] Park S. E. Georgescu A. Huh D. (2019). Organoids-on-a-chip. Science 364 (6444), 960–965. 10.1126/science.aaw7894 31171693 PMC7764943

[B30] Polacheck W. J. Charest J. L. Kamm R. D. (2011). Interstitial flow influences direction of tumor cell migration through competing mechanisms. Proc. Natl. Acad. Sci. U. S. A. 108 (27), 11115–11120. 10.1073/pnas.1103581108 21690404 PMC3131352

[B31] Rizzo G. Bertotti A. Leto S. M. Vetrano S. (2021). Patient-derived tumor models: A more suitable tool for pre-clinical studies in colorectal cancer. J. Exp. Clin. Cancer Res. 40 (1), 178. 10.1186/s13046-021-01970-2 34074330 PMC8168319

[B32] Schindelin J. Arganda-Carreras I. Frise E. Kaynig V. Longair M. Pietzsch T. (2012). Fiji: An open-source platform for biological-image analysis. Nat. Methods 9 (7), 676–682. 10.1038/nmeth.2019 22743772 PMC3855844

[B33] Society A. C. (2023). Key statistics for colorectal cancer [Online]. Available at: https://www.cancer.org/cancer/colon-rectal-cancer/about/key-statistics.html [Accessed 2023].

[B34] van Huizen N. A. Coebergh van den Braak R. R. J. Doukas M. Dekker L. J. M. Jnm I. J. Luider T. M. (2019). Up-regulation of collagen proteins in colorectal liver metastasis compared with normal liver tissue. J. Biol. Chem. 294 (1), 281–289. 10.1074/jbc.RA118.005087 30409905 PMC6322866

[B35] Verduin M. Hoeben A. De Ruysscher D. Vooijs M. (2021). Patient-derived cancer organoids as predictors of treatment response. Front. Oncol. 11, 641980. 10.3389/fonc.2021.641980 33816288 PMC8012903

[B36] Wasson E. M. Dubbin K. Moya M. L. (2021). Go with the flow: Modeling unique biological flows in engineered *in vitro* platforms. Lab a Chip 21 (11), 2095–2120. 10.1039/D1LC00014D 34008661

[B37] Weeber F. van de Wetering M. Hoogstraat M. Dijkstra K. K. Krijgsman O. Kuilman T. (2015). Preserved genetic diversity in organoids cultured from biopsies of human colorectal cancer metastases. Proc. Natl. Acad. Sci. U. S. A. 112 (43), 13308–13311. 10.1073/pnas.1516689112 26460009 PMC4629330

[B38] Zheng Z. Yu T. Zhao X. Gao X. Zhao Y. Liu G. (2020). Intratumor heterogeneity: A new perspective on colorectal cancer research. Cancer Med. 9 (20), 7637–7645. 10.1002/cam4.3323 32853464 PMC7571807

