# Supplementary material for: A perfused multi-well bioreactor platform to assess tumor organoid response to a chemotherapeutic gradient
Source: Front Bioeng Biotechnol. 2023 May 31;11:1193430. doi: 10.3389/fbioe.2023.1193430 (PMC10264793; doi:10.3389/fbioe.2023.1193430)
Supplement: Supplementary file 1 [file DataSheet1.docx]

Supplementary Material

# Supplementary Materials and Methods

## Sacrificial maltodextrin channels

Spray dried maltodextrin was prepared using a 0.4% (w/v) solution of maltodextrin (TapiOK-Organic Tapioca Maltodextrin DE10). The inlet temperature of the spray dryer (Buchi B290) was set to 190°C, outlet temp to (110°C), aspirator to 70%, and flow to 40-45 units. Spray dried maltodextrin was then molded into sacrificial channels using a custom 3D printed mold shown in Supplementary Figure 1 designed to fabricate sacrificial channels that were 1.5 mm in diameter and 15 mm in length.


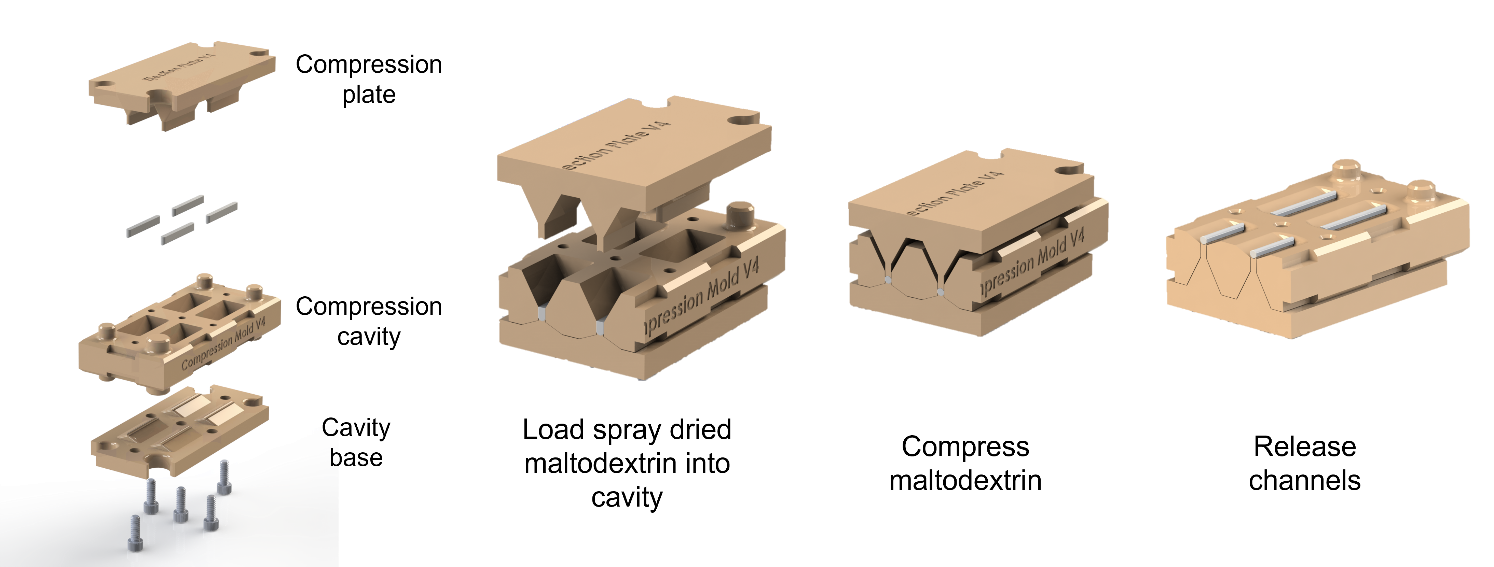


Supplementary Figure 1: Custom mold and process used to compress spray dried maltodextrin into sacrificial channels.

Briefly, the compression cavity and cavity base were connected using 5 x 4-40 screws. 45 g of maltodextrin was weighed out and added to each well of the compression cavity and packed down using a metal spatula. Excess maltodextrin was wiped from the mold and the compression plate was placed on top. The entire assembly was then placed in a hydraulic press with the compression plate on top and pressure was slowly applied until 600 lbf was reached. Compressed maltodextrin channels were then released from the mold by flipping the mold upside down, removing the cavity base, and slowly applying downward pressure until the compression plate pushed the channels out of their wells. Images of molded maltodextrin sacrificial channels were imaged on a microscope (Olympus) and the diameters of the molded channels was measured (1748.6 ± 125.3 μm; n=6).

Compressed maltodextrin channels were connected to the bioreactor inlet and outlet using a 35% (w/w) Pluronic F127 solution and bioreactor hydrogel was cast into each well and allowed to polymerize. The sacrificial maltodextrin was then flushed out, leaving a channel in the hydrogel. Channels that were formed using molded maltodextrin were highly reproducible with average diameter (2271.7 ± 166.5 μm; n=6). The resulting diameter of the channel in the hydrogel may be larger than the measured diameters of the molded maltodextrin due to swelling of the maltodextrin and gel as they become hydrated after casting and flushing. Channel measurements taken after 7 days of flow in the experiment resulted in much smaller channel diameters (454.3 ± 104.2 μm), however these channels were imaged under brightfield, making it more difficult to distinguish the center of the channel where diameter measurements will be larger unlike for fluorescent images.


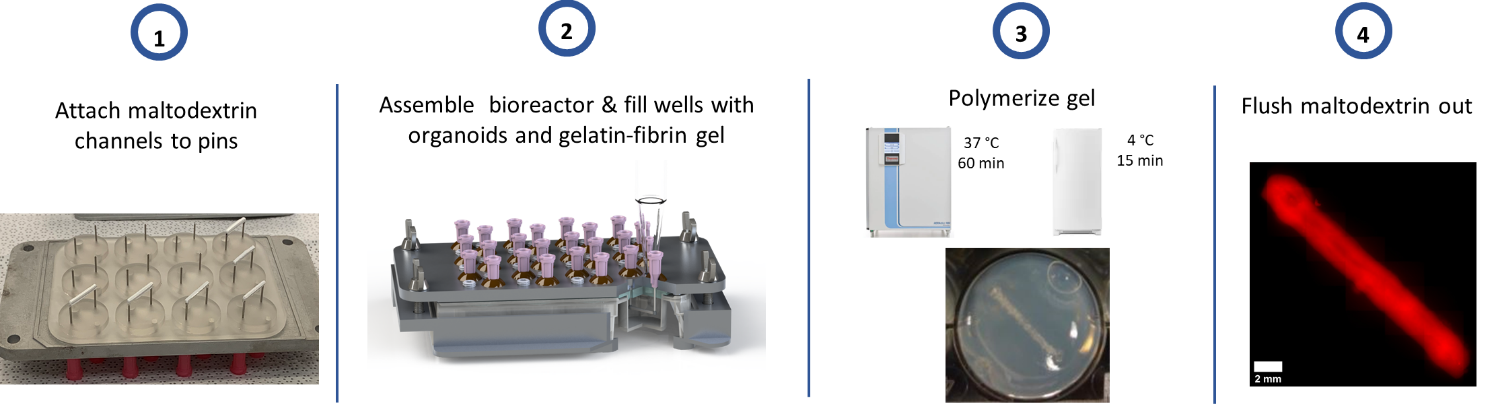


Supplementary Figure 2: Well plate bioreactor setup. (A) Bioreactor lid with PDMS plug gasket and sacrificial channels connected to the bioreactor inlet and outlet pins. (B) Fully assembled bioreactor. (C) Close up of compressed maltodextrin after polymerization of the gel. (D) The resulting formed channel in the bioreactor hydrogel filled with 10 μm fluorescent polystyrene beads for visualization.

## Determination of 5-FU diffusion coefficient used for transport simulations.

Rigorous literature review revealed that the diffusion coefficient of Fluorouracil (5FU) is largely undetermined, especially within non-fluid mediums such as hydrogels. As a result, experimental determination of the diffusivity of 5-FU was necessary before transport simulations for our 12-well bioreactor were conducted. Our experiments were inspired by those described in “*A Method for Determination and Simulation of Permeability and Diffusion in a 3D Tissue Model in a Membrane Insert System for Multi-Well Plates”*; however, deviated significantly in the experimental setup, data sampling methods and methodologies, COMSOL simulations and subsequent analysis.

Diffusion experiments were conducted using hanging 12 mm Transwell inserts (0.4 um pore polycarbonate membranes). 250 μL of uncrosslinked hydrogel were cast onto six membrane inserts. Once cured the samples were placed in contact with 1000 μL of PBS and refrigerated overnight to dialyze any un-crosslinked materials. Membrane inserts were then transferred to a fresh 12 well plate and placed in contact with 1500 μL of fresh PBS (acceptor domain) and 500 μL of 12.5 mM 5-FU in PBS was pipetted on top (donor domain) at T = 0s.

Diffusion timepoints were recorded over an approximate 9-hour period. For each timepoint, acceptor regions were mixed-well prior to removing a 15 μL sample, ensuring a representative average concentration sample was collected. Fluid samples absorbances were then measured using a Nanodrop One at 268 nm. These values were converted to concentrations based using standard curves from similar 5FU + PBS Standards.

Concentration timepoint data from the set of 6 wells were averaged and used in COMSOL simulations to determine the diffusion coefficient of 5FU through our hydrogels. Native COMSOL CAD tools were used to model the relevant geometries. Transport of Diluted Species in Porous Media (tds) and Events (ev) modules were incorporated in our model. The ‘tds’ module was used to model transport of 5FU via diffusion. Simulations used 1e-9 m^2^/s for the diffusion coefficient of 5FU in water, a porosity of 0.9 (determined experimentally) and the impact of insert membranes on small molecule transport, like 5FU, was assumed negligible. The Events (ev) module was used to homogenize the acceptor domain fluid at each sampling timepoint to match the actual experimental process. Iterative parametric sweeps of the diffusion coefficients for 5FU were performed to find the value which minimized the Sum of Least Squares between simulation and experimental datapoints. This process determined a diffusion coefficient of 76.1 um^2^/s for 5FU in our hydrogels.

# Supplementary Results

## Organoid area and circularity

Multiple linear regression was used to test if culture condition and 5-FU dose predict organoid area and circularity. The fitted regression model for organoid area was:

$$Area=0.05923-0.050501*static gel-0.051203*Bioreactor+0.00010966*5-FU dose+0.00011171*static gel*dose+0.00010676*Bioreactor*dose$$

The overall regression was statistically significant (R^2^ = 0.419, F = 51, p = 2.31e-9). Culture conditions are all different, and there is a strong interaction between dose and culture condition.

The fitted regression model for organoid circularity was:

$$Circularity=0.068965+0.18971*Static gel+0.15825*Bioreactor-0.00023981*dose+0.00022672*static gel*dose+0.0001528*bioreactor*dose$$

The overall regression was statistically significant (R^2^ = 0.346, F = 35.7, p = 2.45e-9). Dose did not significantly predict circularity and there was no interaction between dose and culture condition. Culture condition did significantly affect circularity (p<0.001).

**
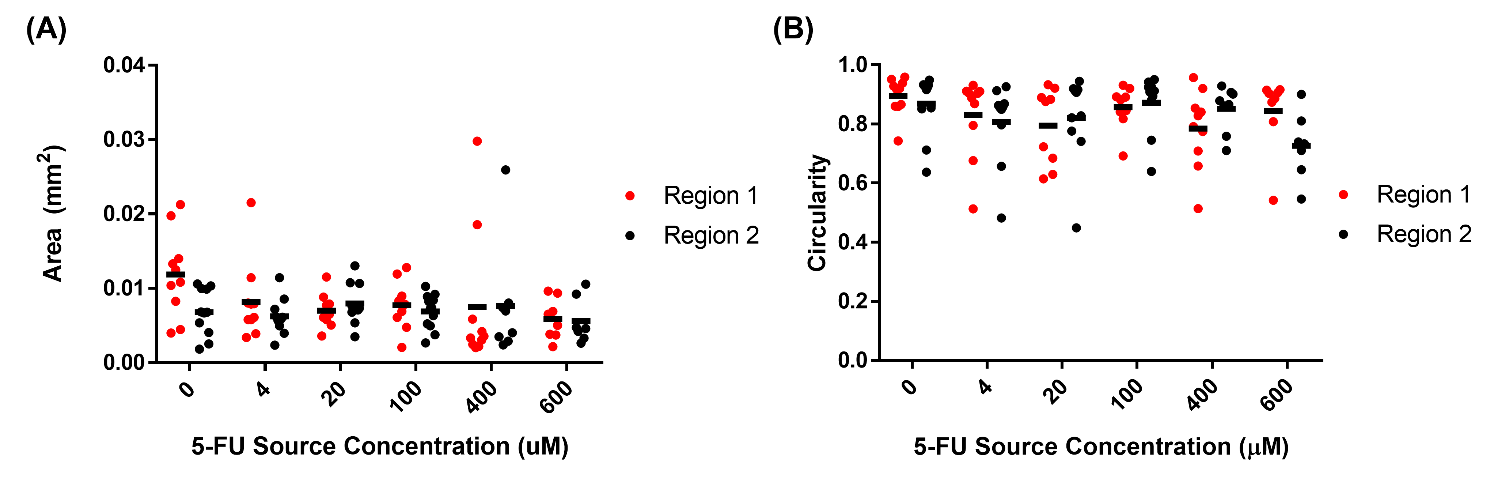
**

**Supplementary Figure 4: Organoid area (A) and circularity (B) for region 1 and region 2 in the bioreactor platform were not statistically significant.**

**Supplementary Figure 5: Lactate-Glo Day 7 EC50 for all culture conditions**

**Supplementary Figure 6: Lactate-Glo and CellTiter-Glo results plotted against applied 5-FU concentration.**
